# Ocular complications in adults with psoriasis: a cross-sectional study in a referral center in Brazil

**DOI:** 10.1007/s10792-024-03147-0

**Published:** 2024-05-05

**Authors:** Ricardo Danilo Chagas Oliveira, Adriano Cypriano Faneli, Dillan Cunha Amaral, Julia Motta Chagas, Jaime Guedes, Ivonise Follador, Maria de Fatima Santos Paim de Oliveira, Bruno F. Fernandes, Luis Claudio Lemos Correia

**Affiliations:** 1 University Hospital Complex Professor Edgard Santos (HUPES) - Federal University of Bahia, Salvador, Brazil; 2 Bahiana School of Medicine and Public Health, Rua Machado Neto, 129, Pituba, Salvador, Bahia CEP 41830-510 Brazil; 3 Faculty of Medicine, Federal University of Rio de Janeiro, Rio de Janeiro, Brazil; 4 Faculty of Technology and Sciences FTC, Salvador, Brazil; 5 Glaucoma Research Center, Wills Eye Hospital, Philadelphia, PA USA; 6 Argumento Institute, Boucherville, Canada

**Keywords:** Eye disease, Psoriasis, Anterior eye segment, Eyelids, Lacrimal apparatus

## Abstract

**Purpose:**

There is limited literature on the ocular manifestations in patients with psoriasis. Therefore, this study aimed to identify the prevalence of and factors associated with ocular manifestations in adults with psoriasis.

**Methods:**

This cross-sectional study included Brazilian adults with psoriasis. The dermatological evaluation included diagnosis, clinical form, Psoriasis Area and Severity Index (PASI) measurement, and location of the lesions. Patients underwent a full ophthalmological examination, including the Schirmer I test, Rose Bengala staining, and tear breakup time tests. The results were analyzed using chi-square and Pearson’s linear correlation tests.

**Results:**

Of the 130 patients assessed, 118 (90.8%) exhibited ocular abnormalities, with meibomian gland dysfunction (MGD) being the most prevalent (59.2%), followed by dry eye disease (DED) (56.2%). A significant correlation was observed between MGD and PASI (*p* = 0.05), and between MGD and certain treatment modalities. DED was significantly associated with PASI (*p* < 0.05). Concurrent use of acitretin was identified as an independent predictor of MGD (odds ratio [OR] = 3.5, *p* < 0.05), whereas PASI was a protective factor against DED (OR = 0.39, *p* < 0.01).

**Conclusion:**

Given the high prevalence of eye disease among individuals with psoriasis, routine ophthalmological assessments are recommended to prevent possible ocular complications.

## Introduction

Psoriasis is a persistent systemic inflammatory condition that is primarily characterized by cutaneous manifestations influenced by genetic and environmental factors through the immune system. It affects approximately 1–3% of the global population; although its onset can occur at any age, its highest incidence occurs among individuals aged 20–30 and 50–60 years. No sex predilection has been observed in previous studies [1].

The exact pathogenesis of psoriasis remains unclear; however, the activation of T cells and heightened pro-inflammatory cytokine activity, particularly that of tumor necrosis factor (TNF)-∂, have received increased attention. In addition to cutaneous lesions, clinical presentations are generally associated with extracutaneous manifestations, including depression, arthritis, uveitis, and cardiovascular conditions. Recent research has also explored potential associations with comorbidities, such as obesity, cancer, hypertension, insulin resistance, dyslipidemia, lymphomas, smoking, and alcoholism [2].

Ocular inflammatory changes are commonly found in patients with immune-mediated diseases, particularly rheumatic diseases such as arthropathic psoriasis (PsA). The mean prevalence of ocular manifestations in patients with psoriasis is approximately 10%, encompassing the adverse ocular effects that result from therapy. These effects can affect various ocular components, such as the optic nerve, retina, cornea, lens, and eyelid, with their incidence varying across studies and different populations [3]. The most commonly reported abnormalities include blepharitis, conjunctivitis, keratitis, dry eye, corneal abscesses, cataracts, orbital myositis, symblepharon, and uveitis [4–6].

Consistent data on the nature and frequency of ocular complications in patients with psoriasis in all clinical forms, particularly within the Brazilian population, are lacking in the literature [7, 8]. Therefore, the present cross-sectional study aimed to determine and document the incidence of ocular manifestations, and analyze their determinants and preventive factors in adults with psoriasis at a referral center in Brazil.

## Materials and methods

### Sample selection

This study enrolled patients assessed at the Psoriasis Clinic within the Dermatology Service at the University Hospital Complex Professor Edgard Santos (C-HUPES/UFBA), a leading center for psoriasis care in Brazil, between November 2012 and June 2014. Participants were selected by the dermatology department, comprising tutors, residents, and service interns who had received prior training in the study protocol.

This study was a cross-sectional investigation, primarily focusing on descriptions and secondarily incorporating analytical aspects. The study was approved by the Institutional Review Board of Federal University of Bahia (No. 33679414.8.0000.0049), and was performed in accordance with the ethical standards outlined in the 1964 Declaration of Helsinki and its later amendments or comparable ethical standards. All participants provided written informed consent willingly and with full awareness.

The exclusion criteria were: (1) the presence of systemic conditions known to potentially induce ocular symptoms, such as connective tissue diseases, systemic vasculitis, sarcoidosis, inflammatory bowel disease, and systemic infectious diseases; (2) a history of ocular trauma or surgery; (3) use of contact lens; and (4) age < 18 years.

### Dermatological assessment

A single researcher and dermatology service tutor provided the initial diagnosis of psoriasis. The extent and severity of the skin lesions were assessed using the Psoriasis Area and Severity Index (PASI), which was categorized as mild to moderate (≤ 10 points) and severe (> 10 points). Data on age, duration since psoriasis diagnosis, time of psoriasis diagnosis, age at diagnosis, body mass index, comorbidities, and therapeutic modalities used during the previous 5 years were collected through a questionnaire administered in person.

### Ophthalmological assessment

All participants were interviewed about their eye symptoms and underwent a complete ophthalmic examination performed by two ophthalmology tutors at the same unit (at different times). The Japanese criteria were applied to establish a diagnosis of dry eye disease (DED), including symptomatic assessment, Schirmer’s I test (Schirmer strips 5 × 35 mm, Ophthalmos, Brazil, São Paulo), tear break-up time (TBUT) test (Fludiag, Oftalmofarma, São Paulo, Brazil), and vital dye staining (Rose Bengal) test (Rose Bengal strips, Ophthalmos, São Paulo, Brazil) [9, 10]. For a definitive diagnosis, the patient must have symptoms and two positive tests. Schirmer’s I test was considered negative when the values were > 10 mm at 5 min [11, 12]. The intensity of Rose Bengal staining was scored up to three points in the cornea and medial and bulbar conjunctiva. TBUT was considered positive when the values were < 10 s in three consecutive measurements [13, 14].

Best-corrected visual acuity, biomicroscopic fundus examination, and Goldman applanation tonometry were performed. The diagnosis of any other eye disease was based on widely used clinical criteria. Crystalline opacity was graded according to Lens Opacities Classification System III. Patients were considered to have glaucoma if they had a previous diagnosis, a history of antiglaucoma medication use, or ocular signs compatible with the disease at the time of examination.

### Data analysis

Descriptive statistics were used to characterize the study population, with categorical variables presented as proportions (with their respective confidence intervals [CIs]) and continuous variables as means and standard deviations. The chi-square test was used to evaluate the possible association between examination abnormalities and patient characteristics. Statistical significance was set at *p* < 0.05. A linear correlation test was performed between the numerical values of the PASI and the number of ocular manifestations. Variables associated with the outcome (*p* < 0.20) were entered into a logistic regression model (multivariate analysis-independent predictor identification). All analyses were performed using SPSS (version 17.0; SPSS Inc., Chicago, IL, USA).

### Sample size calculation

Based on a prior estimate obtained in a pilot study conducted at the beginning of data collection, the prevalence of ocular manifestations in adults with psoriasis was 86%, requiring 129 participants with an accuracy of ± 7%.

## Results

In total, 130 patients were included in this study. Of these, 69 (53.1%; 95% CI 44.1–61.8%) were males, and the mean age was 50.7 ± 13.4 years. Regarding the clinical form of the disease, 115 patients (88.5%; 95% CI 81.6–94.3%) had psoriasis vulgaris, nine had palmoplantar psoriasis (6.9%; 95% CI 3.2–12.7%), two had guttate psoriasis (1.5%; 95% CI 0.19–5.4%), two had pustular psoriasis (1.5%; 95% CI 0.19–5.4%), two had erythrodermic psoriasis (1.5%; 95% CI 0.19–5.4%), and none had an inverted form of psoriasis. The scalp was the most frequent location in 88 (67.7%) patients. Forty patients (30.8%) had been previously diagnosed with psoriatic arthritis. The patient clinical data are presented in Table 1.

**Table 1:** Clinical and epidemiological characteristics of adult patients with psoriasis

Variables	n = 130
	*Mean (SD)*
Age (years)	50.7 (13.4)
Time of disease (months)	140.6 (115.2)
Age at diagnosis (years)	38.9 (15.6)
BMI	27.3 (5.5)
	*n (%)*
Sex	
Male	69 (53.1)
Type of psoriasis	
Vulgaris	115 (88.5)
Palmoplantar	9 (6.9)
Guttate	2 (1.5)
Pustular	2 (1.5)
Erythrodermic	2 (1.5)
Severity criteria	
Scalp lesion	88 (67.7)
Nail lesion	77 (59.2)
Facial lesion	53 (40.8)
Psoriatic arthritis	40 (30.8)
	*Median (Interquartile range)*
PASI (points)	7.9 (3.2–13.3)
	*n (%)*
≤ 10	83 (63.8)
> 10	47 (36.2)

BMI, body mass index; PASI, psoriasis area and severity index; SD, standard deviation

Notably, most individuals (101 patients [77.7%]; 95% CI 69.5–84.5%) had at least one ocular symptom, and 90.8% (118 patients; 95% CI 84.4–95.1%) had at least one sign of an ophthalmological manifestation. The most frequent ocular manifestation was meibomian gland dysfunction (MGD) (77 patients [59.2%]; 95% CI 50.2–67.7%), followed by DED (60 patients [56.2%]; 95% CI 37.3–55.1%). A total of 118 patients had ocular disease (83.1%; 95% CI 75.5–89%). The frequency of ophthalmological diagnoses is presented in Table 2. The most common ocular symptoms related to ocular surface diseases were burning sensation (33.8%; 95% CI 25.7–42.6%), pruritus (30.8%; 95% CI 22.9–38.4%), tearing (29.2%; 95% CI 21.5–37.8%), and foreign body sensation (26.9%; 95% CI 19.5–35.4%).

**Table 2:** Prevalence of ocular manifestations in adult patients with psoriasis compared with that in the general population

Ocular diagnoses	n (%)	95% CI	% General population
MGD	77 (59.2)	50–67	21.2% McCann et al. [15]
Dry eye disease	60 (46.2)	37–55	8.1% McCann et al. [15]
Anterior blepharitis	26 (32.5)	22–43	10–20% Lemp et al. [16]
Cataract	42 (32.3)	24–41	7–20% Malanos et al. [17]
Keratitis	35 (26.9)	19–35	–
Glaucoma	11 (8.5)	4.3–14.6	3.54% Yih-Chung Tham et al. [18]
Low visual acuity	6 (4.6)	1.7–9.7	–
Uveitis	5 (3.8)	1–8	0.2% Dimantas et al. [19]
Scleritis	4 (3.1)	1–7	0.0046% Youning Zhang et al. [20]
Conjunctivitis	1 (0.8)	0–4	–

CI, confidence interval; MGD, meibomian gland dysfunction

There were five cases of uveitis, accounting for 3.8% of all cases (95% CI 1.2–8.7%). All uveitis cases presented with mild bilateral iritis and were associated with concurrent nail and scalp lesions. Two patients were diagnosed with psoriatic arthritis, whereas the remaining three experienced arthralgia. Notably, these three individuals did not meet the diagnostic criteria for psoriatic arthritis, and their severe psoriasis, as reflected by the PASI score, indicated a high disease severity.

There were 11 cases of glaucoma, accounting for 8.5% of all cases (95% CI 4.3–14.6%). All affected individuals were aged > 50 years, with seven men and four women. Of these, only five had a history of corticosteroid use, and only one used topical medication at the time of diagnosis.

The prevalence of cataracts was 32.3% (95% CI 24.3–41%), with the average age of the affected individuals being 61.8 ± 7.8 years. Among these individuals, 36.2% (95% CI 21.5–51.9%) had used topical corticosteroids, and 29.2% (95% CI 15.7–44.5%) had a history of psoriasis treatment, although a clinically significant association was not established.

No association was found between cataracts and phototherapy. The prevalence of cataracts was higher among those who did not undergo phototherapy. Only 9.1% of patients with cataracts (95% CI 2.6–22.6%) had received phototherapy, whereas 34.5% (95% CI 21.5–51.9%) had not.

Regarding the use of retinoids (acitretin), 35.9% (95% CI 21.5–51.9%) of patients with cataracts were taking acitretin at the time of the examination, whereas 30.8% (95% CI 17.6–47%) had no history of acitretin use. The frequency of positive Schirmer’s I test results (< 10 mm in 5 min) was 15.4% (n = 20) and 16.2% (n = 21) in the right and left eyes, respectively. The mean Schirmer’s I test results were 20.2 ± 9.1 mm (range: 2–35 mm) and 19.69 ± 8.8 mm (range: 4–35 mm) in the right and left eyes, respectively. The mean TBUT test results were 9.3 ± 6.5 and 8.5 ± 6.4 in the right and left eyes, respectively. The mean Rose Bengal staining scores were 2.72 ± 1.6 and 3.06 ± 1.7 in the right and left eyes, respectively.

Notably, most patients (92.3%) used at least one form of psoriasis medication. In 20.3% of the cases, two or more therapeutic modalities were concurrently employed, with the most common combination being topical corticosteroids and calcipotriol (12.3%). Methotrexate was the most frequently prescribed systemic medication (44.6%), either as monotherapy or in conjunction with other topical or systemic drugs (Table 3).

**Table 3:** Previous and current treatments for adult patients with psoriasis

Treatment	Current n (%)	Previous n (%)
Topical corticosteroids	72 (55.4)	44 (33.8)
Coaltar	56 (43.1)	21 (16.2)
Methotrexate	50 (38.5)	58 (44.6)
Acitretin	39 (30)	20 (15.4)
Calcipotriol	25 (19.2)	18 (13.8)
Ciclosporin	21 (16.2)	5 (3.8)
Phototherapy	21 (16.2)	11 (8.5)
Inflixmab	3 (2.3)	10 (7.7)
Etarnecept	1 (0.8)	7 (5.4)
Adalimumab	1 (0.8)	1 (0.8)

An initial univariate analysis was performed to examine potential associations of MGD and DED with variables such as sex, presence of facial lesion, presence of scalp lesion, PASI score, and current use of corticosteroids, acitretin, cyclosporin, and infliximab. The results revealed a significant association of MGD with the current use of acitretin (80% vs. 61%, *p* = 0.04) and prior use of calcipotriol (76% vs. 55%, *p* = 0.05), coal tar (73.2% vs. 48.6%, *p* < 0.05), and methotrexate (70% vs. 52%, *p* < 0.05). Furthermore, DED was negatively associated with PASI (31.9% with PASI > 10 and 54.2% with PASI < 10, *p* < 0.05) and prior use of coal tar (57.1% vs. 37.8%, *p* = 0.02). Notably, the Pearson correlation test showed no linear correlation between the PASI numerical values and the number of ocular manifestations, with a correlation coefficient of r = − 0.08 (Table 4).

**Table 4:** Univariate analysis of factors associated with meibomian gland dysfunction and dry eye disease in adult patients with psoriasis

Variables	Meibomian gland dysfunction		Dry eye disease	
	n (%)	*p* value	n (%)	*p* value
Sex		0.067		0.765
Male	46 (66.7)		31 (44.9)	
Female	31 (50.8)		29 (47.5)	
Type of psoriasis		0.245		0.339
Vulgaris	70 (60.9)		51 (44.3)	
Palmoplantar	4 (44.4)		4 (44.4)	
PASI (points)		0.055		0.014
≤ 10	44 (53.0)		45 (54.2)	
> 10	33 (70.2)		15 (31.9)	
Scalp lesion		0.738		0.083
Yes	53 (60.2)		36 (40.9)	
No	24 (57.1)		24 (57.1)	
Nail lesion		0.344		0.869
Yes	43 (55.8)		36 (46.8)	
No	34 (64.2)		24 (45.3)	
Facial lesion		0.190		0.869
Yes	35 (66.0)		24 (45.3)	
No	42 (54.5)		36 (46.8)	
Psoriatic arthritis		0.613		0.333
Yes	25 (62.5)		21 (52.5)	
No	52 (57.8)		39 (43.3)	
Acitretin (current)		0.040		–
Yes	16 (80.0)		–	–
No	61 (55.5)		60 (46.2)	
Calcipotriol (previous)		0.058		0.837
Yes	19 (76.0)		12 (48.0)	
No	58 (55.2)		48 (45.7)	
Coal tar (previous)		0.005		0.029
Yes	41 (73.2)		32 (57.1)	
No	36 (48.6)		28 (37.8)	
Methotrexate (previous)		0.048		0.453
Yes	35 (70.0)		21 (42.0)	
No	42 (52.5)		39 (48.8)	

Variables associated with MGD and DED (*p* < 0.20 and biological plausibility) were included in a logistic regression model to identify independent predictors (Table 5). For MGD, these variables included the presence of scalp lesion (*p* = 0.19), PASI (*p* = 0.05), sex (*p* = 0.06), and current use of infliximab (*p* = 0.16), corticosteroids (*p* = 0.05), acitretin (*p* = 0.04), and cyclosporine (*p* = 0.05). For DED, they were the presence of scalp lesion (*p* = 0.08), PASI (*p* = 0.14), and current use of acitretin (*p* = 0.17). Within this model, the current use of acitretin was independently associated with MGD, with patients using acitretin having a 3.5 times higher likelihood of MGD (odds ratio [OR] = 3.5, 95% CI 1.09–11.17, *p* < 0.05). In addition, a PASI score > 10 (indicating severe psoriasis) was found to be a protective factor against DED, with statistical significance (OR = 0.39, 95% CI 0.18–0.8, *p* < 0.01).

**Table 5:** Logistic regression analysis of independent predictors of meibomian gland dysfunction and dry eye disease in adult patients with psoriasis

Variable	*p*	OR	95% CI
MGD			
Sex	0.06	3.5	1.09–11.1
Scalp lesion	0.19		
PASI > 10	0.05		
Current topical corticosteroids	0.05		
Current acitretin	0.03		
Current cyclosporin	0.05		
Current infliximab	0.16		
DED			
Current acitretin	0.17	0.39	0.18–0.83
Scalp lesion	0.08		
PASI > 10	0.01		

CI, confidence interval; OR, odds ratio; PASI, psoriasis area severity index

## Discussion

Ocular involvement in psoriasis has not been well described in the literature, and most studies have included small sample sizes and populations with specific clinical forms of the disease [4, 7]. In the present study, although the data were collected 10 years ago, a significantly larger sample size of 130 adult patients with all clinical forms of psoriasis from a large referral center in Brazil was used. Consequently, the present study revealed that severe psoriasis, indicated by a PASI score > 10, is a protective factor against DED. Previous studies have shown that DED is more prevalent among adults with psoriasis than among healthy controls; however, the present study is the first to establish a protective correlation between psoriasis severity, indicated by the PASI score, and DED [21–23]. Contrary to previous findings, the present study’s findings revealed that severe psoriasis protects against DED [21, 24].

In the present study, the prevalence of ocular manifestations was 90.8%, which was higher than that reported in the literature. Furthermore, MGD (59.2%) and DED (46.2%) were the most common ocular manifestations in the study population, with a higher prevalence than that in the general population and other published studies on psoriasis. In a recent meta-analysis [15], the prevalence of MGD and DED in the general population were 21.2% and 8.1%, respectively, with some variation across studies [25–27]. In previous studies on adults with psoriasis, the rates of MGD and DED varied from 28–46% to 16–27%, respectively [21–24]. Consistent with the literature, the present study showed that the most commonly reported symptoms, such as burning, itching, tearing, and foreign body sensation, were associated with ocular surface diseases.

In the present study, the prevalence of DED was 46.2%, which is higher than the estimated prevalence of 2.7–19% [28]. DED, in patients with psoriasis, has been investigated as a primary process; however, DED can also occur secondary to other ocular comorbidities, such as chronic conjunctivitis. Impairment of the aqueous component of the tear film is evident in immune-mediated conditions such as psoriasis. The precise pathogenesis of DED and psoriasis remains unclear. In patients with psoriasis, immune-mediated inflammation is initiated by T cells in skin keratinocytes, which results in the development of skin lesions. Notably, prior studies have reported similarities in ocular surface histopathology between psoriasis and the skin, although with reduced parakeratosis [29].

In patients with DED, T cells infiltrate the ocular surface and release specific inflammatory cytokines, which leads to squamous metaplasia of ocular surface epithelial cells and diminished calciform cell differentiation. The association between psoriasis and DED has also been attributed to an L-arginine deficiency and increased β-defensin levels. Psoriatic skin exhibits a reduced concentration of L-arginine, which is characterized by a significant decrease in L-arginine transporter expression within the granulomatous layer of patients with psoriasis. Furthermore, L-arginine deficiency is strongly associated with DED [30].

Prior studies have also highlighted the pivotal role of T helper 17 cells in the pathogenesis of specific ocular surface conditions, with the presence of interleukin (IL)-17 observed not only in systemic autoimmune diseases but also in DED and MGD. IL-17 promotes the secretion of various pro-inflammatory cytokines, including IL-6, TNF-α, IL-1, IL-8, and metalloproteinase-9, by immunological, stromal, and epithelial cells. This cytokine cascade renders individuals with systemic inflammatory disorders more susceptible to ocular diseases [31].

There were no significant associations between the population characteristics (clinical form, duration since diagnosis, and age at diagnosis) and the frequency of eye diseases in the present study, consistent with the findings in previous studies [20]. However, given the small number of clinical types other than psoriasis vulgaris, our findings limit the analysis of these less common clinical types. Furthermore, while there was no linear correlation between the ocular manifestations and disease severity, the present study showed that MGD and DED symptoms were associated with PASI. These results suggest that eye diseases occur in all patients with psoriasis, independent of severity, which is consistent with the findings in the literature [4, 21].

Acitretin use is an independent predictor of MGD. Commonly known ocular effects of retinoids include DED, eye irritation, and blepharoconjunctivitis, although eyelash loss and pseudotumor cerebri syndrome have also been described [3]. This supports the hypothesis that DED in patients with psoriasis may be due to the chronic inflammatory disease itself and/or lacrimal film instability [32]. A previous study reported no association between DED and most therapies, except for an unexpected association with the previous use of coal tar (*p* < 0.05) [4]. While the present study contains no references regarding the side effects associated with topical use of coal tar, we hypothesized that this finding could result from the combined use of coal tar and other drugs without a causal relationship. Robust longitudinal studies are required to test this hypothesis further.

There is an increased risk of uveitis in patients with psoriasis and an even higher prevalence of uveitis in populations with PsA. Among patients with human leukocyte antigen (HLA) B-27, uveitis is more common than other spondyloarthropathies in women [33]. Patients with uveitis have a higher prevalence of HLA-B27, sacroiliitis on magnetic resonance imaging, and ocular surface pathology, and a higher median PsA Impact of Disease Score and Bath Ankylosing Spondylitis Functional Index than those without uveitis [33]. A previous study of 112 patients with psoriatic arthritis reported an iritis rate of 7.1% [34]. Because these patients have a higher risk of developing eye complications, many of which are serious, identifying them is greatly important, particularly for chronic and oligosymptomatic presentations [35]. The lower prevalence of uveitis (3.8%) in the present study population was due to the inclusion of all forms of psoriasis and was not specific to the arthritic form alone.

This study had several limitations. First, the absence of a control group hindered the ability to compare our findings with those of healthy individuals from the same population. Second, the cross-sectional design posed limitations because it precluded the follow-up of the included patients. Thirdly, this experiment only contains a small sample size of patients, and a future study with a larger sample size is still necessary. Finally, the time elapsed since data collection is a constraint in the study.

In conclusion, this study showed that severe psoriasis protects against DED and reports a higher prevalence of eye complications in patients with psoriasis, particularly ocular surface diseases, than previous studies. A better understanding of these manifestations and potential correlations is needed because it provides a theoretical foundation for future epidemiological studies, basic scientific research, and the development of clinical guidelines. There is no consensus regarding the management of these conditions in patients with psoriasis; however, routine eye examination is recommended, particularly in the presence of ocular symptoms. Early detection of subclinical ocular involvement allows prompt initiation of therapy, prevents future complications, and improves patient quality of life.

## Data Availability

The datasets generated during and/or analyzed during the current study are available in the Dryad repository, 10.5061/dryad.q573n5tq0.

## Declarations

### Human and/or animals rights

The study was approved by the Institutional Review Board of Federal University of Bahia (No. 33679414.8.0000.0049), and was performed in accordance with the ethical standards outlined in the 1964 Declaration of Helsinki and its later amendments or comparable ethical standards.
